# Polyhexamethylene biguanide promotes adaptive cross-resistance to gentamicin in *Escherichia coli* biofilms

**DOI:** 10.3389/fcimb.2023.1324991

**Published:** 2023-12-11

**Authors:** Raphaël Charron, Pierre Lemée, Antoine Huguet, Ornella Minlong, Marine Boulanger, Paméla Houée, Christophe Soumet, Romain Briandet, Arnaud Bridier

**Affiliations:** ^1^ Antibiotics, Biocides, Residues and Resistance Unit, Fougères Laboratory, French Agency for Food, Environmental and Occupational Health & Safety (ANSES), Fougères, France; ^2^ Université Paris-Saclay, National Research Institute for Agriculture, Food and the Environment (INRAE), AgroParisTech, Micalis Institute, Jouy-en-Josas, France

**Keywords:** biofilm, biocide, antibiotic resistance, aminoglycoside, biguanide, bacterial adaptation, tolerance, experimental evolution

## Abstract

Antimicrobial resistance is a critical public health issue that requires a thorough understanding of the factors that influence the selection and spread of antibiotic-resistant bacteria. Biocides, which are widely used in cleaning and disinfection procedures in a variety of settings, may contribute to this resistance by inducing similar defense mechanisms in bacteria against both biocides and antibiotics. However, the strategies used by bacteria to adapt and develop cross-resistance remain poorly understood, particularly within biofilms –a widespread bacterial habitat that significantly influences bacterial tolerance and adaptive strategies. Using a combination of adaptive laboratory evolution experiments, genomic and RT-qPCR analyses, and biofilm structural characterization using confocal microscopy, we investigated in this study how *Escherichia coli* biofilms adapted after 28 days of exposure to three biocidal active substances and the effects on cross-resistance to antibiotics. Interestingly, polyhexamethylene biguanide (PHMB) exposure led to an increase of gentamicin resistance (GenR) phenotypes in biofilms formed by most of the seven *E. coli* strains tested. Nevertheless, most variants that emerged under biocidal conditions did not retain the GenR phenotype after removal of antimicrobial stress, suggesting a transient adaptation (adaptive resistance). The whole genome sequencing of variants with stable GenR phenotypes revealed recurrent mutations in genes associated with cellular respiration, including cytochrome oxidase (*cydA*, *cyoC*) and ATP synthase (*atpG*). RT-qPCR analysis revealed an induction of gene expression associated with biofilm matrix production (especially curli synthesis), stress responses, active and passive transport and cell respiration during PHMB exposure, providing insight into potential physiological responses associated with adaptive crossresistance. In addition, confocal laser scanning microscopy (CLSM) observations demonstrated a global effect of PHMB on biofilm architectures and compositions formed by most *E. coli* strains, with the appearance of dense cellular clusters after a 24h-exposure. In conclusion, our results showed that the PHMB exposure stimulated the emergence of an adaptive cross-resistance to gentamicin in biofilms, likely induced through the activation of physiological responses and biofilm structural modulations altering gradients and microenvironmental conditions in the biological edifice.

## 1 Introduction

Antimicrobial resistance (AMR) of bacteria stands as a significant threat to human health in the coming decades. Although resistant strains have existed for millions of years prior to the development of clinical drugs, their (mis)use has accelerated the rapid selection and spread of multidrug resistant bacteria (Davies and Davies, 2010). Among the different bacterial families, concerns on multidrug resistance are particularly high in *Enterobacteriaceae* (Iovleva and Doi, 2017; Alkofide et al., 2020; Atamna-Mawassi et al., 2021). Treatment against *Enterobacteriaceae* include several families of antibiotics, notably fluoroquinolones and aminoglycosides and understanding the mechanisms of bacterial resistance to these antibiotics is of primary importance (Krause et al., 2016; World Health Organization, 2019; Bodendoerfer et al., 2020; Zhu et al., 2020). Additional drivers can also promote the emergence and propagation of AMR in interconnected environments (Aslam et al., 2021), highlighting the importance of identifying these factors and understanding their actual role in AMR. Among these factors, biocides are widely used in various environments, such as food-processing industries to mitigate the risk of pathogenic bacteria dissemination through the food chain. Unlike antibiotics, which often have specific targets in the bacterial cell, biocides typically employ a multi-targeted mode of action. However, mechanisms developed by bacteria to resist biocides can be close or imply common cellular components to those involved in antibiotic resistance and multiple possible cross-resistances have already been identified in different bacterial species (Maillard, 2018; Charron et al., 2023). Schematically, when exposing bacteria to a biocide, cross-adaptations could result from two major types of mechanisms. First, bacteria can become cross-resistant if stable mutations occur in a genetic target involved in both biocide and antibiotic resistance (mutational resistance). Second, physiological adaptations and gene expression modulations in response to biocidal stresses can confer bacteria a greater ability to transiently withstand both biocide or antibiotic challenge (adaptive resistance) (Fernández et al., 2011; Fernández and Hancock, 2012; Sindeldecker and Stoodley, 2021; D’Aquila et al., 2023). The potential of some biocidal active susbtances such as triclosan (Chuanchuen et al., 2001; Sanchez et al., 2005; Karatzas et al., 2007; Tkachenko et al., 2007; Fernández-Cuenca et al., 2015; Lu et al., 2018; Sonbol et al., 2019) or quaternary ammonium compounds (Bore et al., 2007; Fernández-Cuenca et al., 2015; Soumet et al., 2016; Kim et al., 2018; Guérin et al., 2021; Douarre et al., 2022) to select cross-resistance to antibiotics have already been recurrently reported for instance. However, data regarding the antimicrobial resistance effects of biocides are still lacking for the majority of widely used molecules and the underlying mechanisms are unclear. Given their widespread and extensive usage, coupled with the potential for cross-resistance with antibiotics, it is imperative to investigate and comprehend the role of biocides in AMR selection and dissemination.

Numerous studies have examined the impact of biocide on AMR using planktonic bacteria, the most common lifestyle studied in laboratories. However, it is important to recognize that bacteria predominantly live in surface-associated communities, commonly known as biofilms (Römling, 2023). Biofilms represent intricate three-dimensional structures formed by bacterial communities, embedded within a self-produced extracellular matrix. This matrix is composed of a wide variety of substances including exopolysaccharides, lipids, proteins, amyloids and nucleic acids (Karygianni et al., 2020; Flemming et al., 2023). The composition and structure of biofilms depend on the bacterial species and microenvironmental conditions, significantly influencing bacterial metabolism in a reciprocal manner. The dense tridimensional structure creates chemical gradients, resulting in distinct microenvironments for bacteria residing in the periphery, exposed to the external medium, as opposed to those in the inner layers, typically with low access to nutrients and oxygen (Jo et al., 2022). This chemical heterogeneity favors varied physiological responses, giving rise to different populations, including persister cells or viable but non-culturable phenotypes, often characterized by a substantial growth slowdown (Charron et al., 2023). These metabolic states can confer a heightened tolerance to antimicrobials, thus enhancing bacterial survival and potential acquisition of genetic resistance (Levin-Reisman et al., 2017; Nordholt et al., 2021). Furthermore, the external matrix often acts as a barrier, restricting the entry and the diffusion of biocidal substances inside of the biofilm, further increasing bacterial survival (Davison et al., 2010; Bridier et al., 2011).

The combination of these intricate factors is likely to influence significantly how bacteria adapt to their environment. Understanding the potential interplay between biocide exposure, biofilm adaptation and the emergence of AMR is therefore crucial. In this study, we aimed to investigate the effect of the exposure to three commonly used biocidal active substances (benzalkonium chloride (BAC), polyhexamethylene biguanide (PHMB), and N-(3-aminopropyl)-N-dodecylpropane-1,3-diamine (TMN) on the emergence of resistance against two critically important antibiotics for human medicine, the aminoglycoside gentamicin and the fluoroquinolone ciprofloxacin in *Escherichia coli* biofilms (World Health Organization, 2019).

## 2 Materials and methods

### 2.1 Bacterial strains and growth conditions

Seven *E. coli* strains were used in this study. Six strains originated from the collection of the national reference laboratory (NRL) for Antimicrobial Resistance hosted in Anses Fougères laboratory composed of *E. coli* isolated in pork (Ec694; Ec709; Ec723; Ec956) and poultry industries (Ec775; Ec478) between 2014 and 2018. The Ec223 strain originated from the CIRM collection from INRAe and was initially isolated on a chicken. These strains were conserved at -80°C in cryotubes (Mast Group, Bootle, UK). The strains were grown at 37°C on trypticase soy agar (TSA) or in ten times diluted trypticase soy broth (1/10 TSB).

### 2.2 Biocidal substances used and minimal inhibitory concentration assay

Strains susceptibility to three biocides has been tested: BAC (Stepan France); PHMB (Matrix Scientific France) and TMN (Quaron SAS France). The minimal inhibitory concentration (MIC) is here defined as the lowest concentration where cells are unable to grow and were determined as previously described with slight modifications (Guérin et al., 2021). Strains were grown overnight in 1/10 TSB medium and then adjusted to 0.2 (+/− 0.02) optical density at 620 nm (OD620). Microplates were filled with 200 µL of biocides in line A at the highest challenging concentration. 100µL of 1/10 TSB were added in line B to H. A cascade dilution with 100µL of previous line was done from A to H. Finally, 10µL of adjusted bacterial preculture were added in each well. Microplates were incubated at 20°C overnight and MIC were determined after 24 and 48 h of incubation. Experiments were performed in duplicate.

### 2.3 Experimental evolution with three biocides

Strains were grown overnight in 5ml of 1/10 TSB medium from a colony picked on TSA. On the next day, strains were diluted in fresh 1/10 TSB to reach an OD of 0.2 (+/- 0.02). For each strain, 4 columns of 8 wells on a 96-wells flat-bottom microtiter plate (Greiner Bio-One 655161) were filled with 190µL of 1/10 TSB and 10µL of the adjusted subcultures were added to reach a final OD of 0.01 (with an empty column between each). Plates were incubated at 20°C during 1h to enable adhesion of bacteria. Supernatants were then removed and replaced with 200 µl of fresh 1/10 TSB medium and plates were then incubated during 72h at 20°C to enable biofilm development.

After initial biofilm formation, a first cycle began. On the first cycle day, supernatants were removed and 40µL of fresh 1/10 TSB were added. Biofilms were sampled by scratching a quarter of the well bottom surface using tips. 5µL of the biofilm suspension were dropped on TSA plates supplemented with 10 mg/L of the aminoglycoside gentamicin (Gen). This Gen concentration corresponds to 5x the Ecoff value for *E. coli* (https://mic.eucast.org). 5µL of the biofilm suspension were also dropped on TSA supplemented with 1.2 mg/L of the fluoroquinolone ciprofloxacin (Cip). This Cip concentration corresponds to 20x the Ecoff value for *E. coli*. Preliminary tests were done to confirm these concentrations were able to discriminate between a sensitive and a resistant *E. coli* strain. Antibiotic -supplemented TSA plates were then incubated 48 h at 37°C to enable growth of Gen resistant (GenR) and Cip resistant (CipR) variants. Concurrently, 200µL of fresh 1/10 TSB containing biocides were added in wells of the biofilm microtiter plate with each column (8 wells) corresponding to one condition: H_2_O, BAC, TMN, PHMB. The exposure concentrations used were respectively 6.25 mg/L for BAC, 2.5 mg/L for PHMB, 6.6 mg/L for TMN and were chosen based on MIC values determined on planktonic cells of the 7 strains (**Table S1**). Then, used medium was daily renewed with 200 µL fresh 1/10 TSB medium supplemented with biocides for 4 days. On the last day, the used medium was renewed, and the biofilm microtiter plate was incubated 72h at 20°C. The full cycle (one week) was repeated 4 times to reach a total of 28 days of exposure.

### 2.4 Assessment of resistance phenotypic stability

The stability of resistance phenotype in variants collected on antibiotic-supplemented plates after biocide exposure was assessed by repeatedly subculturing resistant variants without selective pressure and then checking the persistence of antibiotic resistance. Concretely, GenR variants collected from biofilms were spread on TSA plates and incubated at 37°C during 24 h. This step was repeated 4 times. Finally, variants were spread a fifth time on Gen-supplemented TSA plates to confirm they still displayed GenR phenotypes.

### 2.5 Enterobacterial repetitive intergenic consensus – PCR

ERIC-PCR was used to compare the patterns observed for the variants and their parental strain to ensure that the different collected variants were not due to contamination as previously described (Cuzin et al., 2021). DNA was first extracted using an InstaGene kit (Bio-rad, Marnes-la-Coquette, France) and amplified using a LightCycler^®^ 480 thermocycler (Roche Diagnostics, Meylan, France) with primers ERIC1-R (ATGTAAGCTCCTGGGGATTCAC) and ERIC2 (AAGTAAGTGACTGGGGTGAGCG) and GoTaq Flexi polymerase (Promega, Charbonnières-les-bains, France) as follows: 95 °CC for 2 min for initial melting; 30 cycles at 95 °CC for 1 min, 54 °CC for 1 min, 72 °CC for 4 min; final extension at 72 °CC for 8 min followed by incubation at 4 °CC. PCR products were then checked on 1% agarose gel and migrated over 90 min at 110 V before being revealed using a GelRED stain (Biotium, Brumath, France).

### 2.6 DNA extraction, sequencing and genomic analyses

*E. coli* genomes were sequenced by paired-end short-reads WGS. Bacteria were first grown on Thermo Scientific™ Tryptone Soya Agar with 5% Sheep Blood plates (Thermo Fischer Scientific, PB5012A), overnight at 37°C. One colony was then inoculated in 5mL of a 1/10 Tryptone Soja Broth (TSB) solution and incubated overnight at 37°C. Total DNA was extracted from 1 ml of bacterial suspension using the Macherey-Nagel Nucleospin Tissue kit according to the manufacturer’s instructions (Macherey-Nagel, Düren, Germany). The concentration and quality of DNA were checked using a BioSpec-nano spectrophotometer (Shimadzu, Marne la Vallée, France). Illumina sequencing was performed on the strains at Institut du Cerveau et de la Moelle épinière (Paris, France) using the Novaseq 6000 SP. Nextera XT kit was used to prepare Illumina libraries. Before analyzing the reads, their qualities were analyzed with FastQC software and filtered with Trimmomatic (v0.39, ILLUMINACLIP : NexteraPE-PE.fa:2:30:10:8:keepBothReads HEADCROP:15 SLIDINGWINDOW:4:25). *De novo* assembly of parental strains was performed with Unicycler (v0.4.8). Contigs smaller than 200nt were removed from the newly formed genomes and Prokka (v1.14.6) was used for annotation. Variant calling was carried out using Snippy (v4.6.0) using filter reads for each variant, using as reference the genome of variant’s parental strain. The analysis pipelines are available on the following GitHub repository: https://github.com/Arnaud-Bridier/BAoBAb

### 2.7 RNA extraction and RT-qPCR

The levels of expression of various genes of interest were also assessed during adaptation to PHMB (list of genes available in **Table S2**). In that aim, Ec223 biofilms were prepared and exposed to H_2_O or PHMB for 28 days as previously described in section 3. At 0 (before exposure), 7, 14, and 28 days, total RNA was isolated from 8 wells for each experimental condition using the NucleoSpin RNA kit according to the manufacturer’s instructions (Macherey-Nagel, Hoerd, France) with the following modifications. A first enzymatic lyse was performed with per well 25 µL mix composed of lysozyme 1 mg/mL, Tris 10 mM and EDTA 1 mM for 10 minutes at 37°C. RNA was quantified, and its purity was assessed with the Biospec-Nano (Shimadzu, Marne la Vallée, France). Reverse transcription, qPCR, primers design and analyses were performed as previously described (Reale et al., 2019) with the following modifications. Reverse transcription was performed with 1.3 µg of total RNA, and quantitative PCR reactions were carried out with 2.6 ng cDNA. All primers were purchased from Sigma-Aldrich (Saint Quentin Fallavier, France), and additional information on oligonucleotide primers are listed in **Table S2**. Using NormFinder software, the gene hcaT was chosen as a reference gene since it did not exhibit any significant variation of expression among the samples. Three independent experiments were performed.

### 2.8 CLSM analysis of biofilm architectural modulations

Biofilm architectures were characterized using CLSM observations in microtiter plates compatible with high resolution imaging (Bridier et al., 2010). For the 7 strains, 72h-biofilms were first grown in 1/10 TSB as previously described and then challenged for 24 h by renewing the used biofilm growth medium with 200µL of 1/10 TSB medium supplemented with 1.25 mg/L of PHMB (or H_2_O for controls). Plates were incubated during 24 h at 20°C. Plates were analyzed with a Leica HCS-SP8 CLSM at the INRAE MIMA2 Imaging Core Facility (Microscopie et Imagerie des Microorganismes, Animaux et Aliments, INRAE, Jouy-en-Josas, doi.org/10.15454/1.5572348210007727E12). Total bacterial cells were dyed using the SYTO™9 green fluorescent permeant nucleic acid stain (Invitrogen, USA) and matrix production was contrasted using 8 µg/ml Congo red staining (Sigma-Aldrich, France). Both markers were excited with an Argon Laser set at 488 nm for SYTO™9 and 514 nm for Congo red. Fluorescence emissions were collected using photomultiplier tubes (PMT) at wavelengths of 500-550nm for SYTO™9 and 600-750nm for Congo red. Biofilm three-dimensional structures were scanned with a 63x water objective lens (numerical aperture: 1.2), with a resolution of 512x512 and a 1 µm Z-step. For each strain, a total of 12 stacks per condition (PHMB-exposed or H_2_O controls) was captured corresponding to 2 Z-stacks per well, with 6 different wells per strain on 2 different plates. Biofilm 3D reconstructions were performed using the Imaris 9.3.1 software (Bitplane, AG-Zürich, Switzerland) via the maximum intensity projection (MIP) section view. Quantitative biofilm biovolumes were extracted from CLSM images with BiofilmQ 0.2.2 (Hartmann et al., 2021).

### 2.9 Statistical analysis

Frequencies of occurrence of GenR variants in H_2_O and in every biocide conditions were compared using the Kruskal-Wallis test (*P* value: * = [0.05-0.01]; ** = [0.01-0.001]; *** = <0.001).

RT-q-PCR data analysis was performed with the Graphpad Prism software. For each week, a Student t-test was performed between the PHMB and H_2_O condition (*P* value: * = [0.05-0.01]; ** = [0.01-0.001]; *** = <0.001).

## 3 Results

### 3.1 Quantification of GenR variants during biofilm exposure to biocides

**Figure 1:**
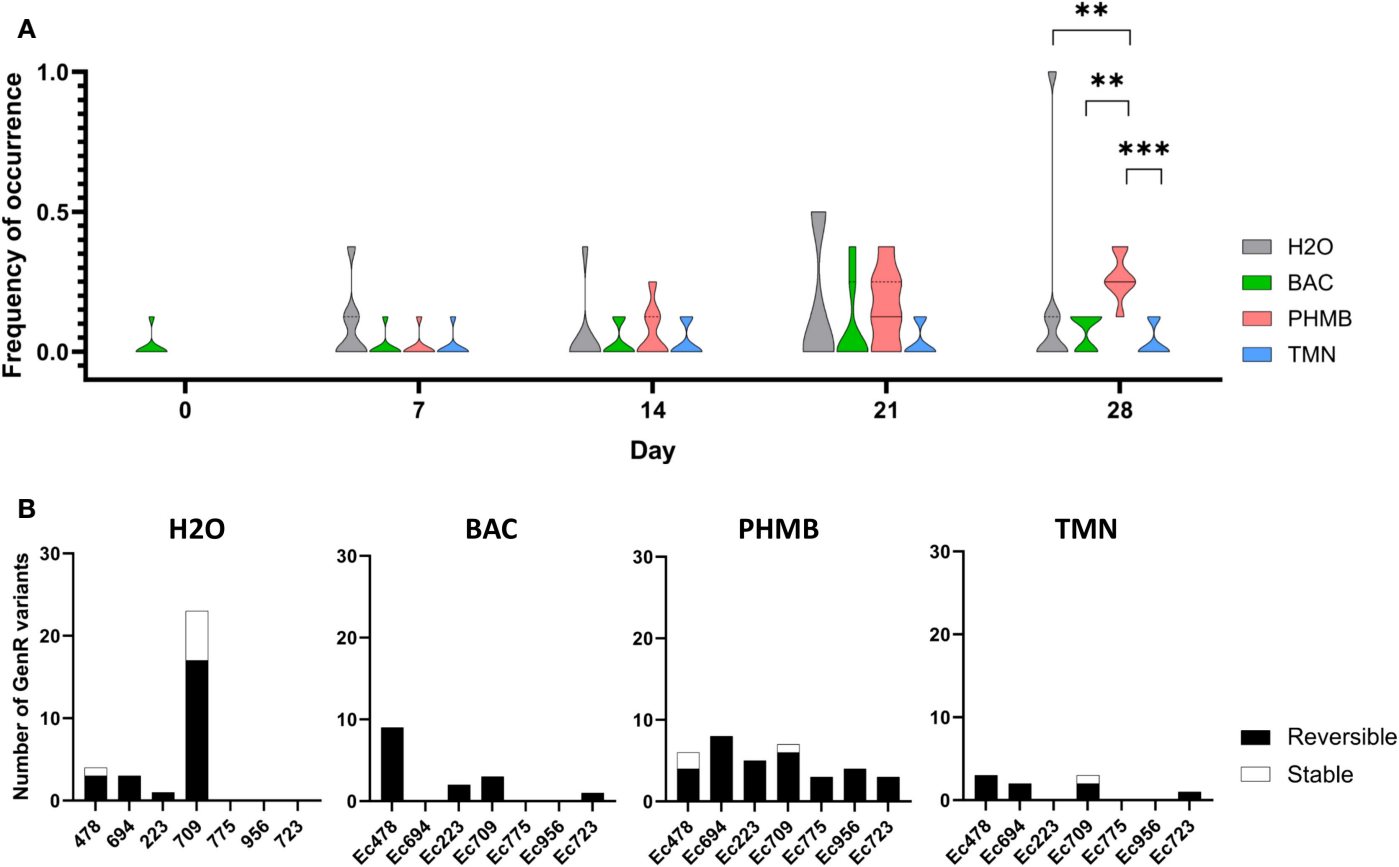
**(A)** Violin plots representing the dynamics of emergence of GenR variants (frequency of occurrence) in biofilms of the different strains in different conditions. Kruskal Wallis Tests were done for each week, comparing every condition, *P* value: *[0.05-0.01]; **[0.01-0.001]; ***<0.001 **(B)** Resistance phenotype stability of GenR variants collected for each of the seven (*E*) *coli* strains during experimental evolution (NB: Due to the high number of variants emerging in H_2_O for the Ec709 strain, a previous selection was made, this graph is thus not showing the real quantity of emerging variants for this specific condition).

*E. coli* biofilms were exposed to BAC, PHMB and TMN for 28 days and bacterial cells collected and enumerated on antibiotic-supplemented TSA plates along biocide exposure to investigate how this stress could influence the emergence of resistant variants against two antibiotics: the fluoroquinolone Cip and the aminoglycoside Gen. We did not detect any resistant variants on Cip-supplemented plates (CipR variants) in the biofilm of the seven *E. coli* strains after exposure to the three different biocides or H_2_O (data not shown). Conversely, Gen resistant (GenR) variants were detected in different strains and conditions with differences between biocides and the control group (H_2_O) as displayed in **Figure 1A**, where the frequency of GenR variant occurrence corresponds to the proportion of replicates for each strain in which at least one GenR variant was detected. After the first week of exposure (day 7), GenR variants tended to emerge preferentially in the control group rather than in presence of biocides (without significative difference). However, upon PHMB exposure, the proportions of GenR-positive replicates variants progressively increased over time in all seven strains when compared to other biocides or in H_2_O controls, revealing a similar effect of PHMB on different *E. coli* strains. After 28 days, the frequency of occurrence of GenR variants was significantly higher in the PHMB-exposed group than in the other conditions, including H_2_O (*p*=0.008704). Conversely, in the H_2_O control, the increase in GenR variants frequency after 28 days was mainly attributed to one strain (Ec709), where GenR variants were seen in every replicate.

Stability assays were performed for each GenR variant to differentiate the number of variants that acquired a stable GenR phenotype and those with a transitory response (**Figure 1B**). Proportions of unstable GenR variants (losing the GenR phenotype after 5 subcultures without Gen selective pressure and called hereafter transient GenR variants) were consistently higher than the stable ones in the different conditions with minor variations. Among the 31 variants isolated in H2O, 7 of them were stable (23%) and emerged in strain Ec478 and especially in strain Ec709. Notably, strain Ec709 was the only strain exhibiting a higher number of GenR variants in the H_2_O control than in the PHMB condition after the 28 days exposure. Concerning the biocides, only 4 of the 60 variants isolated in the different biocide conditions had a stable resistance phenotype, with 3 of them emerging in the PHMB condition (strains Ec478 and Ec709) and another in the TMN condition (strain Ec709).

### 3.2 Genomic analysis of stable GenR variants

The genomes of 11 GenR variants with stable resistance phenotype (8 from Ec709 and 3 from Ec478) that were collected during the experimental evolution phase were sequenced and analyzed in comparison with their respective parental strain. Genetic events responsible for the emergence of GenR phenotype were identified and are displayed in **Table 1**. Genomic analysis of these variants revealed several genetic targets, mainly involved in the electron transport chain. Variants that emerged under biocide conditions exhibited similar affected pathways to those in the H_2_O control group. Two genes were targeted in more than one variant: *cydA* and *atpG*. The most frequently targeted gene was *cydA*, which was mutated in 5 among the 11 variants and was found in both Ec478 and Ec709, as well as in both H_2_O and PHMB conditions. This gene encodes a subunit of cytochrome bd-I, involved in the production of H+, that can be converted into energy for the cell. The *atpG* gene, which codes for a subunit of the ATP synthase, was mutated in 3 out of the 11 variants and was exclusively found in Ec709 strain. Other mutations were identified in similar target genes, including *cyoC*, which codes for another cytochrome subunit, as well as genes associated with ferric transport systems (*fdx* + *fre*), transcription regulators (*rpoS* + arcB), tRNA processing (*tsaB*), glycolysis (*aceE*) and an elongation factor, *fusA*, which is implicated in the translocation of the ribosome during protein synthesis.

**Table 1:** GenR variants emerging in Ec478 and Ec709 in the control group, in PHMB and in TMN throughout the weeks and the associated mutations and aminoacid modifications.

Biocide	Variant name	Gene mutated	Nucleotide modifications	Aminoacid modifications
**H_2_O**	Ec478_H2O_W1	*cydA*	c.173T>A	p.Leu58*
		*ygfZ*	c.423delA	p.Lys141fs
	Ec709_H2O_W1_1	*atpG*	c.728G>C	p.Arg243Pro
	Ec709_H2O_W3_1	*fre*	c.190C>T	p.His64Tyr
		*cydA*	c.444_457delCGGCTGGATGCAAA	p.Asn148fs
	Ec709_H2O_W4_1	*cydA*	c.444_457delCGGCTGGATGCAAA	p.Asn148fs
		Intergenic	c.delT	–
	Ec709_H2O_W4_2	*fusA*	c.2033>T	p.Ala678Val
		*tsaB*	c.484A>G	p.Thr162Ala
	Ec709_H2O_W4_3	*arcB*	c.208G>C	p.Val70Leu
		*cydA*	c.516_520delCGAGC	p.Glu173fs
	Ec709_H2O_W4_4	*aceE*	c.1502G>T	p.Arg501Leu
		*cydA*	c.1284C>A	p.Tyr428*
**PHMB**	Ec709_PHMB_W1	*atpG*	c.728G>C	p.Arg243Pro
		Intergenic	c.G>A	–
	Ec478_PHMB_W2	*cyoC*	c.302G>A	p.Trp101*
	Ec478_PHMB_W4	*fdx*	c.259T>A	p.Cys87Ser
		*rpoS*	c.562A>T	p.Lys188*
		*cydA*	c.331delG	p.Val111fs
**TMN**	Ec709_TMN_W1	*atpG*	c.728G>C	p.Arg243Pro

*, represent the classic annotation to illustrate a "stop codon" resulting in the stop of the aminoacid sequence.

-, not applicable.

### 3.3 Gene expression analysis in presence of PHMB

**Figure 2:**
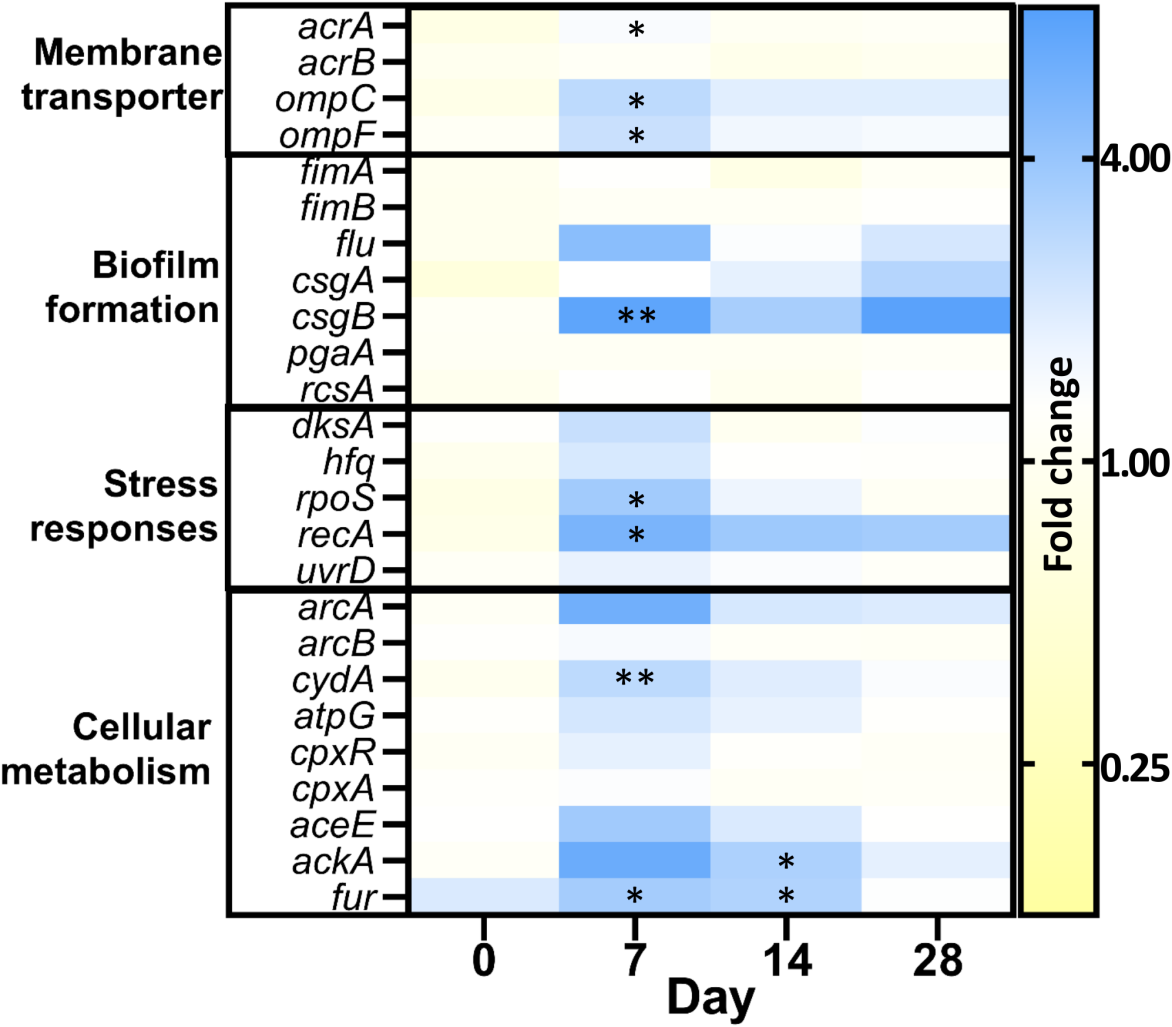
Gene expression differentiation in presence of PHMB, in the Ec223 strain. Fold changes were normalized on the *hcaT* gene and the H2O control. Values >1 indicate an increased expression in presence of PHMB and <1 a decreased expression in presence of PHMB. (*P* value: *[0.05-0.01]; **[0.01-0.001]).

To gain better insights into biofilm adaptation and the bacterial response to PHMB, we examined changes in gene expression levels after 28 days of PHMB-exposure, comparing them to H_2_O condition using RT-qPCR in the Ec223 strain (**Figure 2**). Efflux pumps (*acrAB*) and porins (*ompCF*) were explored because of their known effect against antimicrobials. Biofilm formation genes and stress responses were also investigated because of their relevance in the adaptation in a biofilm model. Finally, cellular metabolism genes were chosen to see if transient resistance was linked to the same genetic targets as the variants with acquired resistance. Globally, most of the pronounced variations occurred after one week of exposure and decreased over subsequent weeks, suggesting an initial response to the PHMB stress followed by a possible adaptation. Various genes were thus significantly overexpressed in the presence of PHMB after 1 week of exposure. Notably, *csgB* (fold change: 7.23) which is involved in curli biosynthesis and biofilm formation, showed a substantial increase, along with genes involved in stress response, such as *rpoS*, the activator of global stress response (fold change: 3.64) and *recA*, which mediates the SOS response (fold change: 5.46). Levels of expression of *csgB* and *recA* in the presence of PHMB remained elevated with regards to fold changes values compared to H_2_O condition during the second part of experimental evolution at days 14 (3.40 and 3.78) and days 28 (7.58 and 3.56), although difference was not statistically significant. In a lesser extent, genes encoding proteins involved in passive transport *ompC/F (*fold changes: *ompC*: 2.77; *ompF*: 2.45) or active efflux *acrA* (fold change: 1.50) were also significantly up-regulated in PHMB compared to H_2_O after 1 week of exposure. Finally, some cell metabolism genes such as *cydA* (fold change: 2.75) and fur (fold change: 3.52), a transcription regulator regulating the expression of several respiratory chain genes and ferric transporters, were also differentially expressed in the first week of PHMB exposure. Other genes showed an overexpression tendency without statistically significant difference, including *arcA*, the global regulator of the cell respiration process, *flu*, the gene involved in the synthesis of the auto-aggregation factor *Ag43*, and other metabolism genes such as *ackA*, an acetate kinase, and *aceE*, a pyruvate dehydrogenase.

### 3.4 Biofilm structural modulation after PHMB exposure

**Figure 3:**
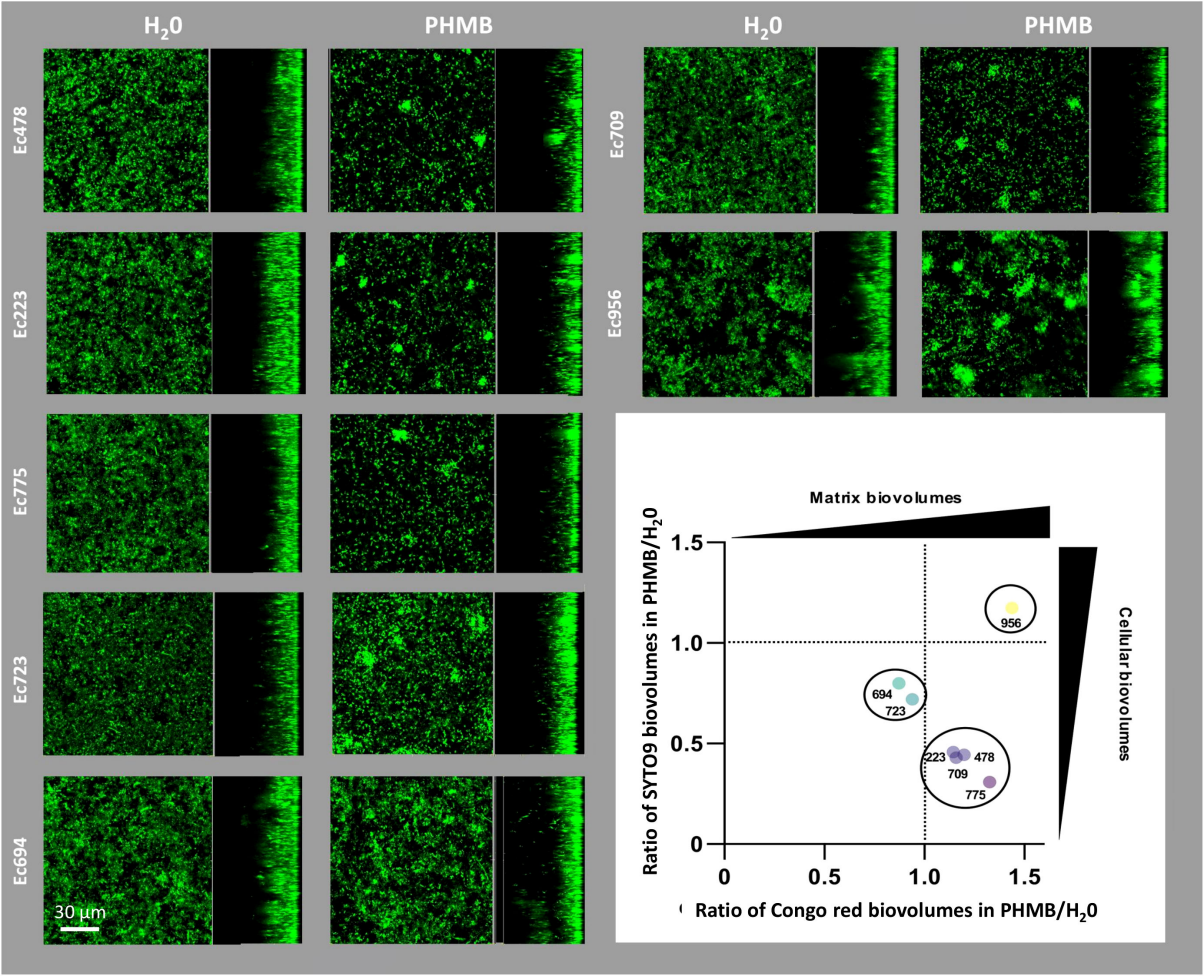
CLSM images of biofilms stained with SYTO™9 in presence or absence of sublethal concentrations of PHMB. On the bottom right, ratios between total cell biovolumes (SYTO™9, y-axis) and biofilm matrix (Congo red, x-axis) of biofilms exposed to PHMB or H_2_O.

The impact of PHMB exposure on the architectural properties of biofilms formed by the seven *E. coli* strains was also investigated using CLSM to observe potential structural perturbations resulting from bacterial community adaptation. Bacterial cells were stained with SYTO™9 and part of the extracellular matrix was stained with Congo red, a dye known to stain amyloid fibers, including curli, which are essential components of the biofilm matrix. First, CLSM observations revealed that PHMB exposure provoked recurrent structural modifications among most *E. coli* strains, leading to the formation of dense clusters as shown on CLSM sections in **Figure 3**. The quantification of biofilm structures following both fluorescent staining (SYTO™9 and Congo red) provided complementary data showing that such architectural modulations in response to PHMB were related to slightly different processes depending on strains. One group composed of Ec223, Ec478, Ec709 and Ec775 had an induction of the matrix production and a reduction in cell biovolume after PHMB exposure. A second group comprising Ec694 and Ec723 also showed a reduction in cell biovolume but no induction of the biofilm matrix. Finally, Ec956 was the only strain with an increase in cell biovolume accompanied by an increase in biofilm matrix production after PHMB exposure.

## 4 Discussion

The escalating threat of antibiotic resistance challenges our understanding of bacterial adaptation mechanisms and the factors contributing to the emergence of resistant bacteria in our environments. Abundant evidence supported the central role of biofilm communities in bacterial adaptation to various antimicrobial substances (Uruén et al., 2020; Usui et al., 2023). However, the underlying mechanisms often remain ambiguous and are highly dependent on the molecules involved. In this contribution, we investigated the impacts of biocide exposures (BAC, TMN and PHMB) on aminoglycoside (Gen) and fluoroquinolone (Cip) resistance emergence in *E. coli* biofilms. Gen and Cip are indeed considered as crucial antibiotics used in the treatment of several gram-negative infections notably involving *Enterobacteriaceae* like *E. coli* (Krause et al., 2016; Zhu et al., 2020).

Experimental evolution experiments revealed here that PHMB exposure resulted in a recurrent increase of transient GenR emergence in the biofilms formed by most of the seven *E. coli* strains compared to H_2_O control. Only one strain, Ec709, showed a reduction in the quantity of variants in PHMB compared to H_2_O (especially due to a high number of GenR variants emerging in H_2_O). Such specific adaptive and mutability features of strain Ec709 would rely on its specific genetic background. Resistant variants appear naturally in bacterial populations and could be preferentially selected in Ec709 by the biofilm lifestyle itself rather than biocidal exposure. This can be related to particular chemical microenvironments in the three-dimensional structure of Ec709 biofilm that could participate in the induction of specific phenotypes and/or selection of genetic variants (Uruén et al., 2020; Charron et al., 2023). In addition, recent findings showed that hypermutator emergence rates are dependent of stress type in *E. coli* populations (Callens et al., 2023). It is therefore possible that in the case of Ec709, the presence of PHMB did not favor variant emergence and/or survival compared to H_2_0 condition.

Conversely, the other biocides BAC and TMN led to a slight decrease of GenR emergence in most *E. coli* strains emphasizing the specificity of exposure effects depending on biocide molecules and their mode of action. Importantly, most variants that emerged under biocidal conditions did not retain the GenR phenotype after removal of antimicrobial stress, suggesting a transient adaptation rather than stable mutation-based resistance. Contrarily to Gen, no Cip variant was detected in any condition during the time of experiment. One assumption is that CipR variants potentially emerging in biofilms were outcompeted here due to a decrease of their ability to survive under biofilm conditions and biocidal exposure. Fluoroquinolones act by inhibiting DNA Gyrase and Topoisomerase IV, enzymes involved in chromosome replication and RNA transcription, and high-level resistance to fluoroquinolones in *E. coli* indeed often requires multiple mutations which are associated with a high fitness cost (Marcusson et al., 2009).

Genomic analysis of the 11 stable GenR variants revealed mutations predominantly affecting the cell respiration pathway and indistinctly selected in H_2_O or upon biocidal stress, showing unspecific evolutionary trajectories. Alteration of genes encoding components of the respiratory and electron transport chains was previously described to be associated with aminoglycoside resistance through the decrease of antibiotic uptake in *E. coli* and other gram-negative species including *Klebsiella pneumoniae* or *Salmonella enterica* for instance (Ibacache-Quiroga et al., 2018; Andersson et al., 2019). The *cydA* gene mutation was especially highly prevalent at the end of the experiment. Interestingly, most of the *cydA* variants had a second mutation, notably in *aceE*, *arcB* and *fre*. These genes are also linked to the cell respiration metabolism, ArcB being known as the main regulator of the cell anoxic respiration process and regulating several of the other mutated genes, like *cydA* (Brown et al., 2022) and *atpG* (Salmon et al., 2005). Moreover, *aceE* has already been associated with a Gen-PHMB cross-resistance mutation (Cuzin et al., 2021). Hence, associations of different mutations in this pathway could strengthen the resistance phenotype and compensate for potential fitness defects caused by the *cydA* mutation. The last *cydA* variant had no other mutated gene, but a mutation in an intergenic region, which could possibly play a role in the fitness of the bacteria. The only variant found in the last two weeks without a *cydA* mutation exhibited two mutations, one in *fusA*, which is also an already-known GenR mutation (Ibacache-Quiroga et al., 2018; Usui et al., 2023) and the other one in *tsaB*. The selection of similar mutations in the H_2_O control and upon biocidal exposure could result from the biofilm lifestyle itself, known to shape specifically adaptive trajectories due to the heterogeneity of microenvironments and associated stress in the three-dimensional structure (Bridier et al., 2017). In line with this, it was recently reported that *E. coli* biofilms upon aminoglycoside treatment favored specific resistance mutations otherwise counter-selected in planktonic cultures, illustrating such a specific biofilm-associated adaptive strategy (Usui et al., 2023).

Interestingly, we observed that PHMB promoted the emergence of transient GenR variants over subsequent weeks of exposure in most of the seven *E. coli* strains. This induction of transient variants upon PHMB exposure could rely on the activation of specific defensive responses to face biocide stress. Coherently, RT-qPCR gene expression analysis revealed the up-regulation of multiple genes linked to metabolic activity or membrane transport in response to PHMB in the first week of exposure, that could illustrate an attempt of bacteria to actively keep the biocide outside the cell. Previous researches reported the upregulation of efflux pumps in response to chlorhexidine digluconate (CHX), a biocide belonging, like PHMB, to the biguanide family (Morita et al., 2003; Fernández-Cuenca et al., 2015; Zhang et al., 2019; Tag ElDein et al., 2021). Interestingly, in another study performed on *Acinetobacter baumannii*, exposure to CHX reduced the resistance of one strain to Gen, while inducing several efflux pumps (Fernández-Cuenca et al., 2015). Here, efflux pumps (AcrA) were indeed slightly impacted by the PHMB pressure in the beginning of exposure. Additionally, general stress responses, like *rpoS*, and the SOS response through *recA* were globally overexpressed, affecting the expression of numerous genes playing a central role in the adaptation to suboptimal conditions and nucleic acid repair (Schellhorn, 2020). It has already been shown that PHMB can bind nucleic acids, which could explain why DNA repair systems are activated (Sowlati-Hashjin et al., 2020). Furthermore, the *csgB* gene involved in curli synthesis, a major amyloid proteinaceous component of extracellular matrix produced by many *Enterobacteriaceae* (Barnhart and Chapman, 2006), showed a prolonged overexpression in the presence of PHMB. Consequently, the denser biofilm matrix due to the overproduction of curli could thereby reinforce the biofilm structure and resistance by limiting PHMB diffusion. The role of curli in biofilm resistance to diverse molecules including biocides and antibiotics was indeed already shown in *E. coli* and other species (Ryu and Beuchat, 2005; Uhlich et al., 2006; Akbey and Andreasen, 2022). CLSM observations of biofilm here confirmed the overproduction of curli fibers after PHMB exposure illustrated by a modulation of the three-dimensional structure and the formation of dense clusters of bacteria associated with an increase in matrix biovolumes compared to H_2_0 controls in most *E. coli* strains tested (**Figure 3**). Interestingly, previous studies have also linked specific subpopulations of cells, also organized into clusters, with increased expression of *csgD*, regulating curli production, and with the production of other matrix components, like cellulose (Grantcharova et al., 2010). The densification of biofilm matrix and reshaping of three-dimensional structure resulting from PHMB-induced curli overproduction could therefore potentially participate to the creation of specific microenvironments (here the clusters) promoting a higher tolerance to Gen notably through slow growth phenotypes (Uhlich et al., 2006; Yan and Bassler, 2019). This could be part of the origin of the increased frequencies of transient GenR variants upon PHMB exposure observed here for the various *E. coli* strains tested.

In this study, Gen variants emerging in biocide conditions mainly exhibited reversible resistance phenotypes. Nevertheless, recent studies suggest a potential link between bacterial tolerance and the facilitated emergence of stable genetic resistances toward antibiotics (Levin-Reisman et al., 2017; Levin-Reisman et al., 2019; Nordholt et al., 2021). An explanation if that tolerant bacteria, capable of surviving longer treatment periods, increase the likelihood of acquiring genetic resistance and often have elevated mutation rates due to the stress they undergo (Windels et al., 2019; Crane et al., 2021). Exposure to PHMB induced stress responses here, such as the SOS response mediated by *recA* and the general stress response involving *rpoS*. The SOS response, typically utilized by bacteria to repair damaged DNA, can lead to the emergence of antibiotic resistant variants due to the use of error-prone polymerases for instance (Crane et al., 2021). As PHMB globally induces physiological responses in biofilms that enhance survival against Gen in most of the *E. coli* strains tested here, it may thereby potentialize the emergence of mutants carrying stables genetic resistance during extended exposures, heightening the risk of antibiotic resistances dissemination and raising public health concerns. Such hypothesis should nevertheless be mitigated regarding the behavior of specific strain as Ec709 here, for which PHMB exposure rather results in a decrease of frequency of GenR emergence, underlining the strain-dependent nature of adaptive strategies.

## 5 Conclusion

We showed in this work that PHMB could indirectly stimulate the emergence of transient Gen resistance in *E. coli* biofilms. Bacterial response to PHMB in biofilms is characterized by the induction of stress responses and architectural modulations, notably through the production of extracellular curlis. These structural rearrangements resulted in the formation of clusters that may constitute specific microenvironments favoring the emergence of slow-growing bacteria with Gen tolerant phenotypes. Overall, this study provided valuable insights into the adaptive trajectories of biofilms exposed to biocidal stress and shed light on potential side effects on bacterial resistance to antibiotics. Further studies should be dedicated to deeply understanding collective biofilm adaptation to biocides and antimicrobial cross-resistance emergence.

## Data availability statement

The datasets presented in this study can be found in online repositories. The names of the repository/repositories and accession number(s) can be found in the article/**Supplementary Material**.

## Author contributions

RC: Conceptualization, Formal analysis, Investigation, Visualization, Writing – original draft, Writing – review & editing. PL: Data curation, Formal analysis, Investigation, Methodology, Software, Writing – original draft. AH: Investigation, Writing – review & editing. OM: Investigation, Writing – original draft. MB: Investigation, Writing – original draft. PH: Investigation, Writing – original draft. CS: Project administration, Resources, Writing – original draft. RB: Conceptualization, Funding acquisition, Supervision, Writing – review & editing, Writing – original draft. AB: Conceptualization, Funding acquisition, Project administration, Resources, Supervision, Visualization, Writing – review & editing, Writing – original draft.
