# Supplementary material for: Polyhexamethylene biguanide promotes adaptive cross-resistance to gentamicin in Escherichia coli biofilms
Source: Front Cell Infect Microbiol. 2023 Dec 11;13:1324991. doi: 10.3389/fcimb.2023.1324991 (PMC10750414; doi:10.3389/fcimb.2023.1324991)
Supplement: Supplementary file 1 [file Table_1.docx]

**Supplementary materials**

**Table S1. Biocide MIC determined for the seven *E. coli* strain (in mg/L).**

|  | **Ec478** | **Ec694** | **Ec223** | **Ec709** | **Ec775** | **Ec956** | **Ec723** |
| --- | --- | --- | --- | --- | --- | --- | --- |
| **BAC** | 12,5 | 12,5 | 12,5 | 12,5 | 12,5 | 6,25 | 12,5 |
| **PHMB** | 2,5 | 2,5 | 5 | 2,5 | 2,5 | 2,5 | 2,5 |
| **TMN** | 13,2 | 13,2 | 13,2 | 13,2 | 6,6 | 13,2 | 6,6 |

**Table S2. Target genes and primers used in this study.**

| **Gene** | **Amplicon length (bp)** | **Sequence 5’ to 3’** | |
| --- | --- | --- | --- |
|  |  | **Forward** | **Reverse** |
| *hcat* | 169 | AGAGAAGTGTCAGCAGTGCC | TGGAGCGTCTGGCTTAAAGG |
| *acrA* | 159 | CGCTTCAGGATAATCCCGCT | CGACAAACAGGCCCAACAAG |
| *acrB* | 171 | TTTTCAGACCCGACGGGAAG | CGAAGATTGAGCTGGGTGGT |
| *ompC* | 116 | CACGGGTCCAGGAGTTGTTT | GCGTCTTGGCTTCAAAGGTG |
| *ompF* | 156 | GCAGTGTCACGCTCGTTTTT | GCTGCCAGAATTTGGTGGTG |
| *fimA* | 121 | GATCCTGGACAGAACGGGTG | GGCCCCGGTTGCAAAATAAC |
| *fimB* | 110 | GAATCCGCTTTCTCGGCAAC | CAAAACCACACGAATGGCGT |
| *flu* | 70 | ACGACATCTGAATACCTGCTACA | TTCGGAGGCAACCACGAAA |
| *csgA* | 100 | GGTCAGGGCTCAGATGACAG | CCGTCATTTCAGAATTTTTGCCG |
| *csgB* | 176 | TATTGATCAGGCGGGCAGTG | GTTGTGTCACGCGAATAGCC |
| *pgaA* | 123 | TCAATGCGCAGTCCCTGATT | CTGGTTACAGGGGCATTCGT |
| *rcsA* | 124 | CTCGACGATATCCTTGGCGA | CCTGACCTGCCATCCACATT |
| *dksA* | 144 | TGCGATCGACTTCATCCCTG | CGTCCCTGAGTATTCTCGCC |
| *hfq* | 82 | ATCACAGTAACAACGCCGGT | TCCTGTTGCGCGGAAGTATT |
| *rpoS* | 107 | GCTCGAACAGCCATTTGACG | CGAAAAAGCGTTGCTGGACA |
| *recA* | 197 | AAGATCAGCAGCGTGTTGGA | CTGGAAATCTGTGACGCCCT |
| *uvrD* | 111 | GACCAGTCAATCTACGGCTGG | GGTAGAGCGGTAGTTTTGCTCC |
| *arcA* | 129 | CGTGCACGAATCGTCAGTTC | GGCGAATGTTGCGTTGATGT |
| *arcB* | 105 | CCAGCAACACCAGGTCGTAT | CGCGTTCTGTGCTGGAAAAA |
| *cydA* | 125 | CGGGATGAAGGCGTACTCTC | GCGTATAGCGTTTCAGCAGC |
| *atpG* | 187 | CCCACGGAGTTGAAGAACGA | TGGAAGACCGCGACGTTAAA |
| *cpxR* | 103 | TGTTTTGCTGTTGCTCGCTC | GGCGCAGATGACTATCTCCC |
| *cpxA* | 76 | AGTAACGCCGTACCCAGTTG | AGCAGCGTCTGCTTTCTGAT |
| *aceE* | 88 | TGCTTTCCTGGAAGGTCGTC | GTGCGGATAGGAAGAGAGGC |
| *ackA* | 185 | GGTCTGACCGAAGTGACCAG | GCGGCATTTTCACCGATACC |
| *fur* | 193 | TCGTGGTGATGTTGCTGTGT | TTCTTCAGGAGCCGGACAAC |

**Table S3. Biosample and Sequence Read Archive (SRA) accession numbers of parental strains and GenR variants from NCBI. Accession numbers of genome assemblies were also included for parental strains used as references for mutations detections.**

| **Strain name** | **Biosample accession** | **SRA accession** | **Genome assembly accession** |
| --- | --- | --- | --- |
| Ec223 | [SAMN34162446](https://www.ncbi.nlm.nih.gov/biosample/SAMN34162446) | [SRX19950532](https://www.ncbi.nlm.nih.gov/sra/SRX19950532%5baccn%5d) | [ASM3193264v1](https://www.ncbi.nlm.nih.gov/datasets/genome/GCF_031932645.1/) |
| Ec478 | [SAMN33867613](https://www.ncbi.nlm.nih.gov/biosample/SAMN33867613) | [SRX19762823](https://www.ncbi.nlm.nih.gov/sra/SRX19762823%5baccn%5d) | [ASM3216290v1](https://www.ncbi.nlm.nih.gov/datasets/genome/GCF_032162905.1/) |
| Ec694 | [SAMN33867245](https://www.ncbi.nlm.nih.gov/biosample/SAMN33867245) | [SRX19763326](https://www.ncbi.nlm.nih.gov/sra/SRX19763326%5baccn%5d) | [ASM3216292v1](https://www.ncbi.nlm.nih.gov/datasets/genome/GCF_032162925.1/) |
| Ec709 | [SAMN33867612](https://www.ncbi.nlm.nih.gov/biosample/SAMN33867612) | [SRX19763732](https://www.ncbi.nlm.nih.gov/sra/SRX19763732%5baccn%5d) | [ASM3216296v1](https://www.ncbi.nlm.nih.gov/datasets/genome/GCF_032162965.1/) |
| Ec723 | [SAMN33867235](https://www.ncbi.nlm.nih.gov/biosample/SAMN33867235) | [SRX19761680](https://www.ncbi.nlm.nih.gov/sra/SRX19761680%5baccn%5d) | [ASM3216294v1](https://www.ncbi.nlm.nih.gov/datasets/genome/GCF_032162945.1/) |
| Ec775 | [SAMN33867606](https://www.ncbi.nlm.nih.gov/biosample/SAMN33867606) | [SRX19763817](https://www.ncbi.nlm.nih.gov/sra/SRX19763817%5baccn%5d) | [ASM3216300v1](https://www.ncbi.nlm.nih.gov/datasets/genome/GCF_032163005.1/) |
| Ec956 | [SAMN33867237](https://www.ncbi.nlm.nih.gov/biosample/SAMN33867237) | [SRX19763879](https://www.ncbi.nlm.nih.gov/sra/SRX19763879%5baccn%5d) | [ASM3216302v1](https://www.ncbi.nlm.nih.gov/datasets/genome/GCF_032163025.1/) |
| Ec478_H2O_W1 | [SAMN34509072](https://www.ncbi.nlm.nih.gov/biosample/34509072) | [SRX20202989](https://www.ncbi.nlm.nih.gov/sra/SRX20202989%5baccn%5d) | - |
| Ec478_PHMB_W2 | [SAMN34509073](https://www.ncbi.nlm.nih.gov/biosample/34509073) | [SRX20202990](https://www.ncbi.nlm.nih.gov/sra/SRX20202990%5baccn%5d) | - |
| Ec478_PHMB_W4 | [SAMN34509074](https://www.ncbi.nlm.nih.gov/biosample/34509074) | [SRX20202991](https://www.ncbi.nlm.nih.gov/sra/SRX20202991%5baccn%5d) | - |
| Ec709_H2O_W1_1 | [SAMN34509085](https://www.ncbi.nlm.nih.gov/biosample/34509085) | [SRX20203307](https://www.ncbi.nlm.nih.gov/sra/SRX20203307%5baccn%5d) | - |
| Ec709_PHMB_W1 | [SAMN34509093](https://www.ncbi.nlm.nih.gov/biosample/34509093) | [SRX20203316](https://www.ncbi.nlm.nih.gov/sra/SRX20203316%5baccn%5d) | - |
| Ec709_TMN_W1 | [SAMN34509094](https://www.ncbi.nlm.nih.gov/biosample/34509094) | [SRX20203308](https://www.ncbi.nlm.nih.gov/sra/SRX20203308%5baccn%5d) | - |
| Ec709_H2O_W3_1 | [SAMN34509087](https://www.ncbi.nlm.nih.gov/biosample/34509087) | [SRX20203310](https://www.ncbi.nlm.nih.gov/sra/SRX20203310%5baccn%5d) | - |
| Ec709_H2O_W4_1 | [SAMN34509089](https://www.ncbi.nlm.nih.gov/biosample/34509089) | [SRX20203312](https://www.ncbi.nlm.nih.gov/sra/SRX20203312%5baccn%5d) | - |
| Ec709_H2O_W4_2 | [SAMN34509090](https://www.ncbi.nlm.nih.gov/biosample/34509090) | [SRX20203313](https://www.ncbi.nlm.nih.gov/sra/SRX20203313%5baccn%5d) | - |
| Ec709_H2O_W4_3 | [SAMN34509091](https://www.ncbi.nlm.nih.gov/biosample/34509091) | [SRX20203314](https://www.ncbi.nlm.nih.gov/sra/SRX20203314%5baccn%5d) | - |
| Ec709_H2O_W4_4 | [SAMN34509092](https://www.ncbi.nlm.nih.gov/biosample/34509092) | [SRX20203315](https://www.ncbi.nlm.nih.gov/sra/SRX20203315%5baccn%5d) | - |
